# The Effect of Faking on the Correlation Between Two Ordinal Variables: Some Population and Monte Carlo Results

**DOI:** 10.3389/fpsyg.2018.01876

**Published:** 2018-10-12

**Authors:** Marco Bressan, Yves Rosseel, Luigi Lombardi

**Affiliations:** ^1^Department of Psychology and Cognitive Science, University of Trento, Rovereto, Italy; ^2^Department of Data Analysis, Ghent University, Ghent, Belgium

**Keywords:** Pearson correlation, Spearman correlation, sample generation by replacement (SGR), fake ordinal/discrete data, population analysis, Monte Carlo simulations

## Abstract

Correlational measures are probably the most spread statistical tools in psychological research. They are used by researchers to investigate, for example, relations between self-report measures usually collected using paper-pencil or online questionnaires. Like many other statistical analysis, also correlational measures can be seriously affected by specific sources of bias which constitute serious threats to the final observed results. In this contribution, we will focus on the impact of the fake data threat on the interpretation of statistical results for two well-know correlational measures (the Pearson product-moment correlation and the Spearman rank-order correlation). By using the Sample Generation by Replacement (SGR) approach, we analyze uncertainty in inferences based on possible fake data and evaluate the implications of fake data for correlational results. A population-level analysis and a Monte Carlo simulation are performed to study different modulations of faking on bivariate discrete variables with finite supports and varying sample sizes. We show that by using our paradigm it is always possible, under specific faking conditions, to increase (resp. decrease) the original correlation between two discrete variables in a predictable and systematic manner.

## 1. Introduction

The relations between variables is at the heart of psychological research. Correlation is a statistical index representing the degree to which two variables vary together and reflects their strength of association. Although correlations do not imply causation, in many behavioral studies, empirical hypotheses are tested in terms of simple associations or, eventually, lack of them.

There are many well-known sources of bias that are serious threats to the measuring of empirical correlations. For example, the presence of measurement errors in the data does not allow to directly observe the true association between the variables. In Classical Test Theory (CTT, Lord, 1980), the observed measurement is understood as the sum between the true unknown value and its measurement error. In general, the larger the measurement error, the poorer (in terms of reliability) the correlation estimates are. Another widely accepted assumption in CTT states that the measurement error is normally distributed with zero mean and unknown variance. Under this assumption, an analyst can easily construct confidence intervals to quantify the uncertainty of estimated correlations. However, this approach may be questionable whenever the observations are corrupted by more complex sources of noise such as, for example, asymmetric errors or structured errors.

An additional important threat for correlations, is the presence of outliers in the data. Outliers can interfere in the measurement process by either increasing or attenuating the observed correlations (Anscombe, 1973). In this situation, one can use specific data analysis procedures (e.g., graphical, statistical, distance, and density based approaches) to try to mitigate the effect of outliers or to identify and eventually remove them from the analysis. Robust procedures can be applied to evaluate if extreme observations significantly impact on the overall result of correlational analyses by attenuating (resp. boosting) their values (Wilcox, 2016).

Also the treatment of ordinal variables as if they were continuous variables (a common practice in many psychological studies) constitutes a potential threat to the correct estimation of correlational measures. Similarly, the splitting of a continuous variable in a countable number of discrete categories can certainly bias the final estimated correlation. This phenomenon is called “broad categories” or “few ordinal category” and has been extensively studied in the psychometric literature (e.g., Pearson, 1913).

Moreover, in some particular contexts such as, for example, educational and employment selection, researchers have access to data only from a restricted population (range restriction bias) and yet must attempt to estimate parameters for the target unrestricted population. Some classic solutions have been proposed in the statistical literature to mitigate this bias on the basis of specific correction formulas (e.g., Spearman's correction of attenuation, Kelley's correction for range restriction, Spearman, 1904; Kelley, 1947).

Last but not least, correlation analysis may be affected by the presence of fake observations in the data. This aspect is particularly relevant for researchers working with self-report measures collected in sensitive scenarios such as for example, risky sexual behaviors and drug addictions (e.g., Furnham, 1986; Zickar and Robie, 1999; McFarland and Ryan, 2000) where individuals tend to fake their responses in order to meet strategic goals (e.g., avoiding being charged with a crime, Mittenberg et al., 2002; Hall and Hall, 2007; Ziegler et al., 2012). In this context, the core problem is that there is no basis to assume that participants are responding honestly, nor is there an easy way to verify the validity of answers, or a robust methodology to detect the presence of fake responses in the observed data (Lombardi and Pastore, 2016). Like for the outlier threat, the presence of fake observations in the sample can also artificially increase (resp. decrease) the strength of association between two variables (Ellingson et al., 1999; Zickar and Robie, 1999; Pauls and Crost, 2005; Ziegler and Buehner, 2009; Galić et al., 2012).

In this contribution, we will focus on this last kind of bias and explore the impact of the fake data threat on the modulation of sizes or strengths of correlational results. In particular, the fake data problem entails a crucial question: If data includes fake observations, to what extent will the empirical correlation be different from what it actually is? In other words, *which* percentage of fakers (within a target sample) and *what* type of faking response process would jointly lead the results (e.g., correlation estimation, model fit evaluations) to be somehow different from what they actually are? In order to answer these questions we adopted a quantitative approach which uses an effect size measure based on the Cohen's *Q* statistic between correlations (Cohen, 1988).

The psychometric literature about modeling faking data is now growing and covers many aspects, data analysis oriented and applicative ones. For example, the issue of fake data has been investigated using *ad hoc* empirical paradigms such as ad lib faking or coached faking to collect data and simulate fake reports (Zickar and Robie, 1999; Zickar et al., 2004). In particular, in the last decade some authors have proposed rational methods for assessing fake data in social desirability contexts or faking-motivating situations by using factor analytic approaches (Ferrando, 2005; Ferrando and Anguiano-Carrasco, 2009, 2011) as well as factor mixture models (Leite and Cooper, 2010). However, less attention has been paid to some more general aspects related to the impact of faking on the observed statistical results. For example, how sensitive are the observed statistics to possible fake data? Are the statistical results still invariant under one or more scenarios of faking manipulations? In this contribution we will adopt a statistical approach, called Sample Generation by Replacement (SGR, Lombardi and Pastore, 2012), to analyze uncertainty in inferences based on possible fake data as well as to evaluate the implications of fake data for correlational results.

In particular, in this contribution we adapted the SGR representation to allow the study of the effect of faking on a target correlation statistic both at the population and sample levels. The latter approach will also be useful to understand the impact of faking on the correlations under varying sample size conditions and how they departure from the corresponding population ones. Overall the fake data threat problem will be tested using two widespread correlation indices (Pearson correlation and Spearman correlation) computed on two ordinal variables with varying levels of categories and different typologies of faking models.

To anticipate our results, we show that by using the SGR paradigm it is always possible, under certain faking conditions, to either increase or decrease the original correlation between two ordinal variables in a predictable and systematic manner. From an applied perspective, this general result is at the same time interesting and alarming as it may show how a statistically significant correlation could easily be the effect of a false positive association due to spurious (or inflated) correlations that may be elicited from structured faking manipulations. By contrast, nonsignificant correlations may reflect true associations which have been masked because the observations have been perturbed by some destructuring faking process.

The remainder of the article is organized as follows: the first section starts with a brief recapitulation about the two correlation indices used in the context of discrete variables. Next, the article continues by illustrating the main components of the SGR approach (as originally introduced by Lombardi and Pastore, 2012) followed by the novel population adaptation. The next section evaluates the effect of faking on the population correlation. We continue by presenting a Monte Carlo simulation study about the evaluation of how the coefficient of correlation can change under different fake data perturbations and different sample sizes. Finally, the discussion section presents conclusions regarding the main theoretical findings of our study together with some relevant comments about limitations, potential new applications and extensions of the SGR approach.

## 2. Measures of correlation

Two correlation measures were examined in this study: the product moment correlation (Pearson's correlation) and the Spearman rank-order correlation. Although other types of correlation indices may be adopted in the evaluation of the strength of association between two ordinal variables (e.g., Kendall's Tau-b, Somers's, Gamma statistic, Agresti, 2013) we wanted to understand the impact of fake manipulations for the two most widely used measures of associations known today. In our context, the discrete version of the parametric product moment correlation will be used as a reference (suboptimal) model against which we will compare a more suitable nonparametric measure of association for ordinal variables (the Spearman's correlation). In what follows, we will introduce the main terms and notation to describe the two correlation models in the context of discrete random variables with finite support.

Let *X* and *Y* be two purely discrete random variables with a common finite support^1^ {1, 2, …, *v*} with *v* ∈ ℕ. Moreover, let *p*_*ij*_ be the joint discrete probability distribution *P*(*X* = *i, Y* = *j*) (with *i, j* = 1, …, *v*), and let *p*_*i*._ and *p*_.*j*_ be the corresponding marginal probabilities. The discrete cumulative marginal distribution functions are computed as $F_{i}=\sum_{k\;=\;1}^{i}p_{i.}$ and $G_{j}=\sum_{sk=\;1}^{j}p_{.j}$ for *X* and *Y*, respectively. Finally, let (**x**, **y**) = ((*x*_1_, *y*_1_), …, (*x*_*n*_, *y*_*n*_)) be the bivariate sample of *n* observations drawn from the discrete population distribution. Now we are in the position to describe the discrete versions of the two correlation models.

### 2.1. Pearson's product moment correlation

The parametric Pearson's correlation measures the linear dependence between two *continuous variables* (Pearson, 1895). However, in applied research it is common practice to use it also with discrete numeric variables (e.g., Likert-type items) by treating the ordinal values as interval-based values. In an ideal context, the relation between the two variables is linear and deviations from the straight line model generally attenuates the magnitude of the correlation. In addition, the two variables are assumed to be normally distributed and homoscedastic. Unfortunately, in the discrete variable setting the application of the product moment correlation does not meet several of these basic requirements. In the present contribution, we studied the behavior of the parametric correlation in the discrete setting and its departure from the optimal continuous context using different finite discrete supports (e.g., *v* = 2, 5, 7) for the observed variables.

#### 2.1.1. Sample correlation

The discrete version of the Pearson's correlation, denoted by *r*, can be computed according to the following formula:

(1) $$r=\frac{{\hat{\sigma }}_{xy}}{{\hat{\sigma }}_{x}{\hat{\sigma }}_{y}}\;=\;\frac{\sum_{s\;=\;1}^{n}\left(x_{s}-\bar{x}\right)(y_{s}-\bar{x})}{\sqrt{\sum_{s\;=\;1}^{n}{\left(x_{s}-\bar{x}\right)}^{2}}\sqrt{\sum_{s\;=\;1}^{n}{\left(y_{s}-\bar{y}\right)}^{2}}}$$

where ${\hat{\sigma }}_{\text{x}\text{y}}$ is the sample covariance, and ${\hat{\sigma }}_{\text{x}}$ and ${\hat{\sigma }}_{\text{y}}$ are the sample standard deviations. Finally, $\bar{x}$ and $\bar{y}$ indicate the two sample means for the discrete samples **x** and **y**, respectively.

#### 2.1.2. Population correlation

At the population level, the Pearson's coefficient is usually denoted by the Greek letter ρ and defined as follows:

(2) $$\begin{array}{l} \rho =\frac{\sigma_{xy}}{\sigma_{x}\sigma_{y}}\\ \;=\frac{\left[\sum_{i\;=\;1}^{v}\sum_{j\;=\;1}^{v}ijp_{ij}\right]-\left[\sum_{i\;=\;1}^{v}ip_{i.}\right]\left[\sum_{j\;=\;1}^{v}jp_{.j}\right]}{\sqrt{\sum_{i\;=\;1}^{v}i^{2}p_{i.}-{\left[\sum_{i\;=\;1}^{v}ip_{i.}\right]}^{2}}\cdot \sqrt{\sum_{j\;=\;1}^{v}j^{2}p_{.j}-{\left[\sum_{j\;=\;1}^{v}jp_{.j}\right]}^{2}}} \end{array}$$

where σ_**xy**_, and σ_**x**_, σ_**y**_, are the population covariance and the two standard deviations, respectively.

### 2.2. Spearman's rank correlation

The Spearman correlation coefficient is a nonparametric measure of association between two variables which is based on ranks and is one of the earliest measures of correlation to be developed in the statistical literature (Spearman, 1904). It requires that both variables be measured in at least an ordinal scale in such a way that the observations in **x** and **y** can be ranked in two ordered sets. The main assumption of the Spearman correlation is that the two variables must be monotonically related to each other.

#### 2.2.1. Sample correlation

At the sample level, the Spearman's correlation is generally described by the following formula:

(3) $$r=\frac{{\hat{\sigma }}_{rank(x,y)}}{{\hat{\sigma }}_{rank(x)}{\hat{\sigma }}_{rank(y)}}=\frac{\sum_{s\;=\;1}^{n}\left(R_{s}-\bar{R}\right)(S_{s}-\bar{S})}{\sqrt{\sum_{s\;=\;1}^{n}{\left(R_{s}-\bar{R}\right)}^{2}\cdot \sum_{s\;=\;1}^{n}{\left(S_{s}-\bar{S}\right)}^{2}}}$$

where *R*_*s*_ and *S*_*s*_ are the ranks of observations *x*_*s*_ and *y*_*s*_, whereas $\bar{R}$ and $\bar{S}$ are the two sample rank averages.

#### 2.2.2. Population correlation

The population version of the Spearman's correlation for variables with discrete and finite supports has been characterized in the statistical literature only recently (Nešlehová, 2007):

(4) $$\rho =\frac{3\sum_{i=\;1}^{v}\sum_{i=\;1}^{v}p_{ij}\left[\left(F_{i}+F_{i-1}\right)\left(G_{j}+G_{j-1}\right)-1\right]}{\sqrt{\left(1-\sum_{i=\;1}^{v}p_{i.}^{3}\right)\left(1-\sum_{j=\;1}^{v}p_{.j}^{3}\right)}}$$

The sample version of formula (4) can be easily obtained by replacing the population terms *p*_*ij*_, *p*_*i*._, *p*_*j*._, *F*_*i*_, and *G*_*j*_ with the corresponding sample estimates based on the *v* × *v* contingency table derived from {**x**, **y**}. It can be proved (see Nešlehová, 2007) that the sample version of formula (4) reduces to the well known sample formula (3) when *X* and *Y* are discrete random variables with finite supports.

## 3. Sample generation by replacement, SGR

### 3.1. Standard SGR

The SGR methodology is characterized by a two-stage sampling procedure which uses two distinct models to simulate the process of faking. The first model serves to generate synthetic data before any kind of fake data corruption. This data generation process reflects how ideal data should behave if they were fake-observation free. The second model is a data replacement process which mimics the perturbation carried out by the faking observations. The main idea is that the mechanism of faking can be understood as a process which transforms the original stream of information into a new stream reflecting the final corrupted message. In the standard SGR approach, the first procedure is realized by means of basic Monte Carlo (MC) techniques, whereas the second procedure is modeled by adopting *ad-hoc* probabilistic models (e.g., Lombardi and Pastore, 2012, 2014; Pastore and Lombardi, 2014). In the present work we will use the SGR framework to study the population behavior of correlation statistics under several scenarios of faking. In particular, Cohen's effect size measure in the context of bivariate correlations for ordinal variables will be explored in detail. Moreover, to better highlight the population-level analysis of the faking problem we will slightly modify the standard SGR notation by introducing a novel matrix representation.

Let (*X*^*d*^, *Y*^*d*^, *X*^*f*^, *Y*^*f*^) be a tuple of discrete variables with the same common support {1, 2, …, *v*}. In the SGR representation, the four variables can be partitioned into two groups defining the honest/uncorrupted condition, {*X*^*d*^, *Y*^*d*^}, and the faking condition, {*X*^*f*^, *Y*^*f*^}, respectively. The joint probability distribution for the honest condition is represented as follows:

(5) $$\begin{array}{rcl} p_{ij}^{d} & = & P\left(X^{d}=i,Y^{d}=j|\theta_{d}\right) \end{array}$$

with (*i, j*) ∈ {1, 2, …, *v*}^2^ and where *θ*_*d*_ is the parameter array associated with the uncorrupted model. By contrast, the faking condition is represented by means of a conditional distribution

(6) $$\begin{array}{rcl} z_{hk|ij} & = & P\left(X^{f}=h,Y^{f}=k|X^{d}=i,Y^{d}=j,\theta_{f}\right) \end{array}$$

with (*i, j, h, k*) ∈ {1, 2, …, *v*}^4^ and where *θ*_*f*_ is the parameter array associated with the faking model. Formula (6) identifies the so called *replacement distribution* in a SGR model. This distribution represents the conditional probability of replacing the original observed values (*i, j*) in the uncorrupted model with the new fake values (*h, k*) and constitutes the main kernel of any SGR representation (Lombardi and Pastore, 2012). The joint probability distribution for the faking condition is, therefore, the marginal probability

(7) $$\begin{array}{rcl} z_{hk} & = & \overset{v}{\underset{i=1}{\sum }}\overset{v}{\underset{j=1}{\sum }}p_{ij}^{d}z_{hk|ij} \end{array}$$

A further simplifying assumption in the SGR framework requires the replacement distribution to meet the *conditional independence* property:

(8) $$\begin{array}{rcl} z_{hk|ij} & = & z_{h|i}^{1}z_{k|j}^{2} \end{array}$$

with $z_{h|i}^{1}$ and $z_{k|j}^{2}$ being the two separate conditional distributions $P\left(X^{f}=h|X^{d}=i,\theta_{f_{X}}\right)$ and $P\left(X^{f}=k|X^{d}=j,\theta_{f_{Y}}\right)$, respectively. Therefore, Equation (7) reduces to

(9) $$\begin{array}{rcl} z_{hk} & = & \overset{v}{\underset{i=1}{\sum }}\overset{v}{\underset{j=1}{\sum }}p_{ij}^{d}z_{h|i}^{1}z_{k|j}^{2} \end{array}$$

Note that the two conditional distributions are characterized by different parametrizations, *θ*_*f*_*X*__ and *θ*_*f*_*Y*__. This reflects the idea that the replaced values are only governed by the corresponding original (uncorrupted) values and the specific faking process. Moreover, the faking process can be different for the two variables *X* and *Y* depending on the values of the parameters *θ*_*f*_*X*__ and *θ*_*f*_*Y*__. For example, we can decide to adopt two different directions and intensities of faking when a respondent uses different faking strategies for the two items/variables *X* and *Y*.

The marginal distribution of the faking component (9) can be described in a compact form using the following matrix notation:

(10) $$\begin{array}{rcl} \text{Z} & = & {\left({\text{P}}^{T}{\text{Z}}_{1}\right)}^{T}{\text{Z}}_{2} \end{array}$$

with $\text{P}=\left[p_{ij}^{d}\right]$ being the *v* × *v* matrix representing the joint distribution for the honest condition, and ${\text{Z}}_{1}^{T}=\left[z_{h|i}^{1}\right]$ and ${\text{Z}}_{2}^{T}=\left[z_{k|j}^{2}\right]$ being the two *v* × *v* transpose matrices associated with the replacement distributions, respectively. Formula (10) can be used to sample bivariate observations which are in line with the faking model parameterized according to (*θ*_*d*_, *θ*_*f*_*X*__, *θ*_*f*_*Y*__).

### 3.2. Mixture SGR

In some empirical circumstances the model assumption that all participants are equally faking their responses may be simply unrealistic. In general, it seems more useful to belief that while some individuals tend to manipulate their responses, others may simply provide clean honest responses. To this purpose, the SGR representation can be easily extended to allow each respondent to be part of one of two separate groups: (a) an honest group (b) a faking group. If a participant belongs to the first group, the corresponding responses will be sampled according to model **P**. By contrast, if a participant belongs to the faking group, the responses will be sampled on the basis of the faking model **Z**. In more formal terms, the mixture SGR representation takes the following form

(11) $$\begin{array}{rcl} s_{xy} & = & \left(1-\alpha \right)p_{xy}+\alpha z_{xy},\;\left(x,y\right)\in {\left\{1,2,\ldots ,v\right\}}^{2} \end{array}$$

where *s*_*xy*_ is the mixture probability of the observed variables *X* = *x* and *Y* = *y*. In Equation (11), parameter α ∈ [0, 1] denotes the probability weight in the mixture model and represents the proportion of fakers in the population. In matrix notation, Equation (11) takes the following form:

(12) $$\begin{array}{rcl} \text{vec}\left(\text{S}\right) & = & \left(1-\alpha \right)\text{vec}\left(\text{P}\right)+\alpha {\text{Z}}_{2}^{T}⊗{\text{Z}}_{1}^{T}\text{vec}\left(\text{P}\right) \end{array}$$

with vec(·) and ⊗ being the vectorization operator and the Kronecker product, respectively. Note that special instances of the mixture model are obtained when *α* = 0 (resp. *α* = 1). In this particular case, the joint probability distribution **S** reduces to the honest model **P** (resp. faking model **Z**).

#### 3.2.1. Models of honest responses

In general, several options are available to modeling the joint distribution **P** for honest responses of discrete variables with finite supports (e.g., Samejima, 1969; Muthén, 1984; Jöreskog and Sörbom, 1996; Moustaki and Knott, 2000). In the SGR framework a natural choice is the adoption of a multivariate latent variable representation named underlying variable approach (UVA, Muthén, 1984; Jöreskog and Sörbom, 2001). The basic idea of this approach is that the two observed discrete/ordinal variables are treated as metric through assumed underlying bivariate normal variables (*W*_1_, *W*_2_). In the UVA context the parametrization *θ*_*d*_ of the joint distribution **P** is an array containing a set of *v*−1 thresholds that are used to discretize the two underlying continuous variables and a correlation parameter ρ_*d*_ that modulates their linear relationship. More precisely, the UVA parametrization is given by

(13) $$\begin{array}{l} p_{ij}^{d}=\int_{\xi_{i-1}}^{\xi_{i}}\int_{\xi_{j-1}}^{\xi_{j}}\phi (w_{1},w_{2}|0,R_{d})dw_{1}dw_{2},\\ \;i=1,\ldots ,v;j=1,\ldots ,v \end{array}$$

where ξ_1_, …, ξ_*v*−1_ are the *v*−1 thresholds (with ξ_0_ = −∞, ξ_*v*_ = +∞) and *ϕ* is a bivariate standardized distribution with mean **0** = (0, 0) and correlation matrix **R**_*d*_ with correlation parameter ρ_*d*_.

#### 3.2.2. Models of fake responses

Nowadays there is a broad consensus that faking is an intentional response distortion aimed at achieving a personal gain (e.g., MacCann et al., 2012). In this study, we will limit our scope to two relevant scenarios of faking: (a) faking good (b) faking bad. Faking good can be defined as a conscious attempt to present false information to create a favorable impression with the goal of influencing others (e.g., Furnham, 1986; Zickar and Robie, 1999; McFarland and Ryan, 2000). In general, fake good respondents are able to modify their scale scores by providing more extreme response values (e.g., Furnham, 1986; Viswesvaran and Ones, 1999; McFarland and Ryan, 2000; Griffin et al., 2004). In the SGR context a fake-good manipulation always represents a context in which the responses are exclusively subject to positive feigning:

$$\begin{array}{l} X^{f}>X^{d}\;\left(\text{resp. }Y^{f}>Y^{d}\right). \end{array}$$

For example, faking good manipulations could be associated to purchased evaluations by an online shop to grow up in the ranking. Reversely, faking bad indicates the conscious attempt to create a less positive impression by providing lower response values:

$$\begin{array}{l} X^{f}<X^{d}\;\left(\text{resp. }Y^{f}<Y^{d}\right). \end{array}$$

For example, in a selection of compulsory military service a candidate may try to fake a personality inventory to mimic some mental disease with the aim to avoid the service.

**Figure 1:**
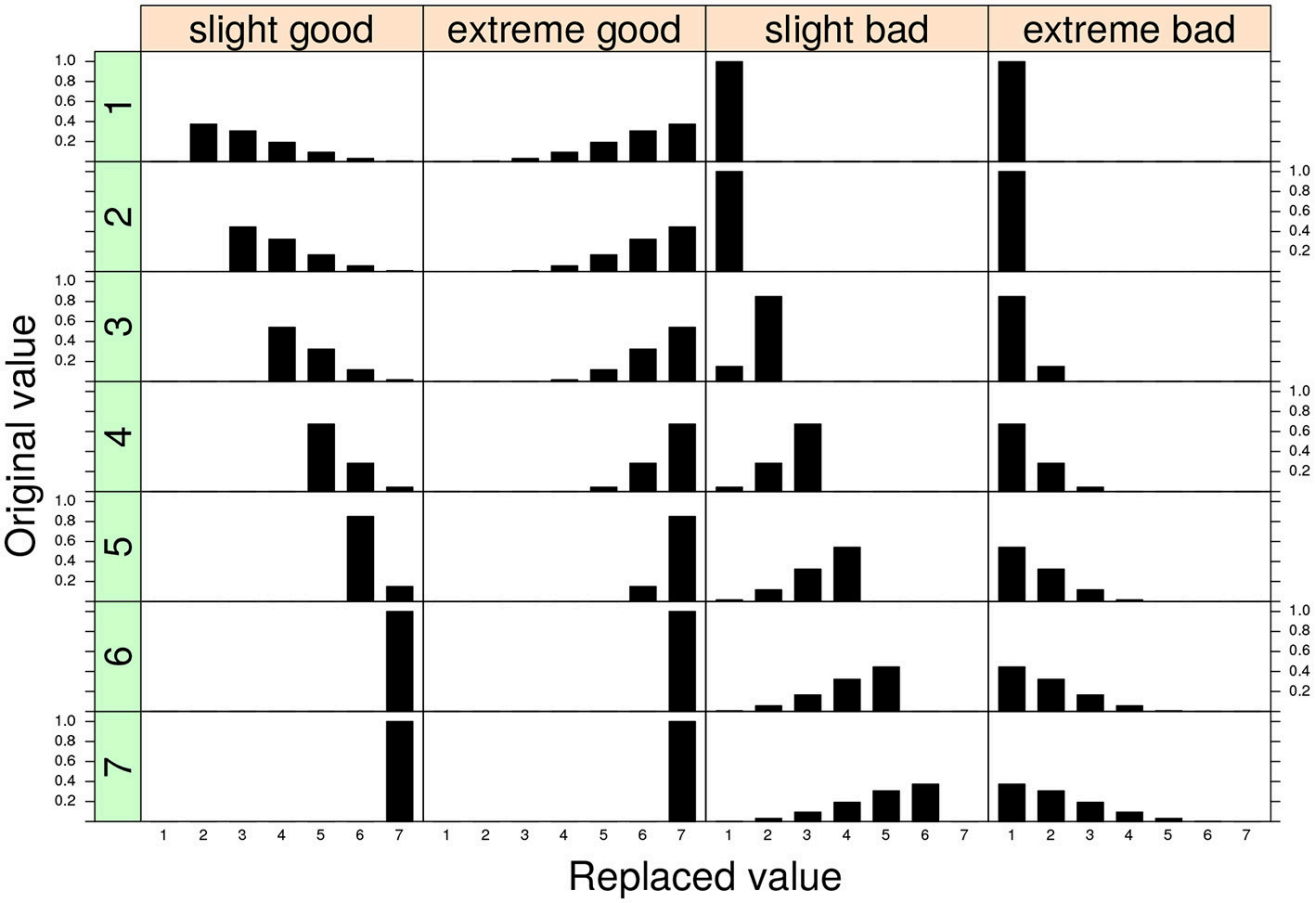
Four examples of replacement distributions for a 7-point discrete variable. Each column in the graphical representation corresponds to a different conditional replacement distribution. Each row in the graphical representation corresponds to a different original 7-point discrete value. The parameter assignments for the four models are: *slight* model (*γ* = 1.5, *δ* = 4) and *extreme* model: (*γ* = 4, *δ* = 1.5). The replacement distributions are applied for the two configurations *faking good* and *faking bad*, respectively.

In the SGR approach the faking mechanism is captured by the replacement models **Z**_1_ and **Z**_2_ parametrized according to a discrete version of the generalized beta distribution with shape parameters *θ*_*f*_*X*__ = (*γ*_*X*_, *δ*_*X*_) and *θ*_*f*_*Y*__ = (*γ*_*Y*_, *δ*_*Y*_), respectively (see Lombardi and Pastore, 2012, 2014). In general, this model parametrization is very flexible and can easily characterize the two typologies of faking manipulations with varying levels of intensity. In particular, if we set the values of the shaping parameters as 1 ≤ *γ* < *δ* ≤ 5, we can reconstruct replacement distributions which mimic mild positive shifts in the value of the original observed response (Figure 1, first column). This configuration can be applied whenever we believe that the original observations have been corrupted by a slight faking good process (Zickar and Robie, 1999; Zickar et al., 2004).

By contrast, the condition 1 ≤ *δ* < *γ* ≤ 5 describes a faking scenario in which the fake value corresponds to an extreme shift in the original value (extreme model, Figure 1 second column). The extreme model can be adopted if we believe that the original observations have been corrupted by a sort of extreme faking good process (Zickar and Robie, 1999; Zickar et al., 2004). Note that a totally symmetric representation can be straightforwardly constructed for the faking bad condition and will not be discussed further here (see also Figure 1, third and fourth columns). For additional details the reader may refer to the original works about the model parametrization in the SGR framework (e.g., Lombardi and Pastore, 2012, 2014).

## 4. Population evaluation of fake correlations

### 4.1. The cohen's *Q* statistic

In order to evaluate the impact of faking on the population correlations we adopted an effect size measure based on the Cohen's *Q* statistic (Cohen, 1988). Of course, an alternative and legitimate perspective would instead require to study and analyze the impact of faking on the significance levels of the correlational results. However, in the present contribution we preferred to limit the analysis on the *Q* measure as we were mainly interested in representing the effect size modulations of faking on the correlational statistic which has a clear meaning at the population as well as sample level. Nonetheless, in the discussion session we will briefly return on the possibility to provide an alternative significant level analysis of the faking problem.

At the population level, the effect size *Q* is defined as the difference between two Fisher transformations of the population correlation (ρ_*m*_) computed on the basis of the mixture joint distribution **S** and the population correlation (ρ_*d*_) of the original uncorrupted joint distribution **P**:

(14) $$\begin{array}{r} Q=\frac{1}{2}\log\frac{1+\rho_{m}}{1-\rho_{m}}-\frac{1}{2}\log\frac{1+\rho_{d}}{1-\rho_{d}} \end{array}$$

We calculated the *Q* statistic for the two correlation indices (Pearson and Spearman) separately using the corresponding population formulas (Equations 2, 4).

**Figure 2:**
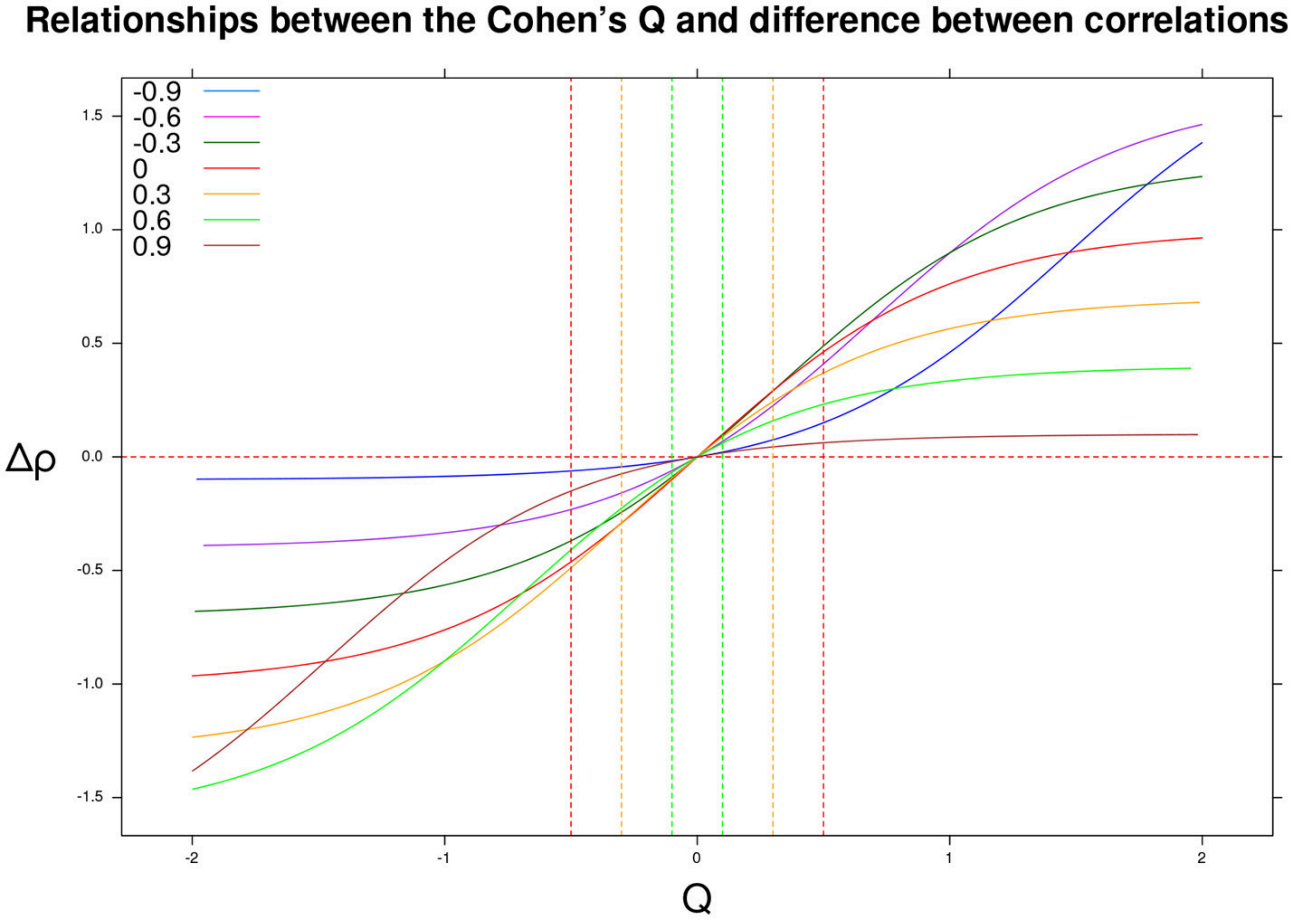
Differential correlation Δρ = ρ_*m*_−ρ_*d*_ as a function of the Cohen's *Q* statistic and original correlation ρ_*d*_ ∈ {−0.9, −0.6, −0.3, 0, 0.3, 0.6, 0.9}. The vertical lines identify negative (resp. positive) small, medium, and large effect sizes.

Cohen (1988) also provided substantive interpretations for different ranges of effect size values. A *small* effect size, *Q* = 0.10, corresponds to small differences between the uncorrupted correlation and the mixture correlation, (e.g., ρ_*d*_ = 0.20, ρ_*m*_ = 0.29). A *medium* effect size, *Q* = 0.30, is linked with mild differences, (e.g., ρ_*d*_ = 0.20, ρ_*m*_ = 0.46). Finally, a *large* effect size, *Q* = 0.50, corresponds to wider differences in the correlations, (e.g., ρ_*d*_ = 0.20, ρ_*m*_ = 0.61). Figure 2 shows the association between *Q* and the differential correlation Δρ. In particular, if the *Q* statistic is positive (resp. negative), then the differential correlation Δρ = ρ_*m*_−ρ_*d*_ is also positive (resp. negative).

### 4.2. Results of the population analysis

**Figure 3:**
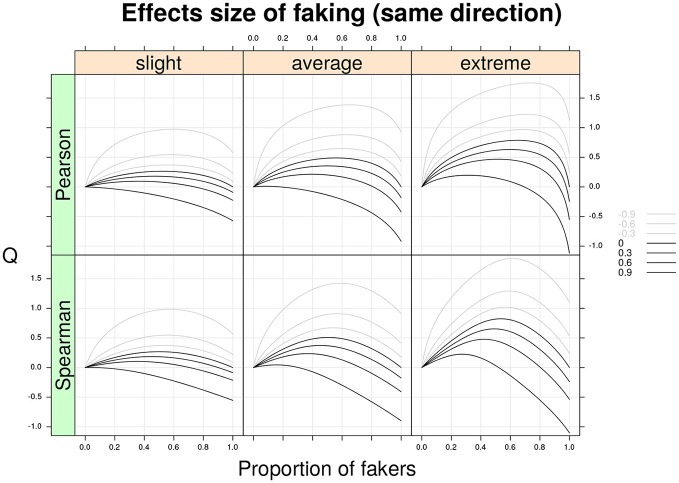
Cohen's *Q* statistic as a function of proportion of fakers in the population, type of faking model (slight, average, extreme), and original true correlation. The population-level analysis regards the two correlation indices (Pearson and Spearman) under the same model and direction of faking applied on two discrete variables with common finite support (*v* = 5) and symmetric marginal distributions with mass probabilities 0.06, 0.25, 0.38, 0.25, and 0.06, respectively. Note the average model (*γ* = 3, *δ* = 3) represents a compromise between the slight model and the extreme model.

Figure 3 shows the Cohen's *Q* statistic (computed for Spearman and Pearson correlations separately) as a function of proportion of fakers and original correlation ρ_*d*_ for three models of faking (slight, average, and extreme) under the assumption that both variables *X* and *Y* are subjected to the same direction of faking (either faking good or faking bad). As expected, larger effect sizes are associated with extreme faking models and higher proportions of fakers in the mixture population. In general, the Cohen's *Q* statistic may take either positive or negative values depending on the specific faking model and proportion of fakers in the mixture population. For example, under a slight faking model and 50% of fakers in the mixture population, an original Spearman correlation, ρ_*d*_ = −0.60, is affected from a bias of *Q* = 0.54. Similarly, under an extreme faking model with 20% of fakers in the mixture population, an original zero Spearman correlation shows a faking bias of *Q* = 0.45. Moreover, if we consider a very high positive original correlation, ρ_*d*_ = 0.90, with also 20% of fakers in the population this results into a bias of *Q* = 0.2. By contrast, for the same correlation a larger proportion of fakers (e.g., 70%) boils down to a negative bias *Q* = −0.33. Even for smaller proportions of fakers (e.g., lower than 10%), the effect of faking can definitively have an impact on the original correlation ρ_*d*_ (e.g., Figure 3, bottom-right).

**Figure 4:**
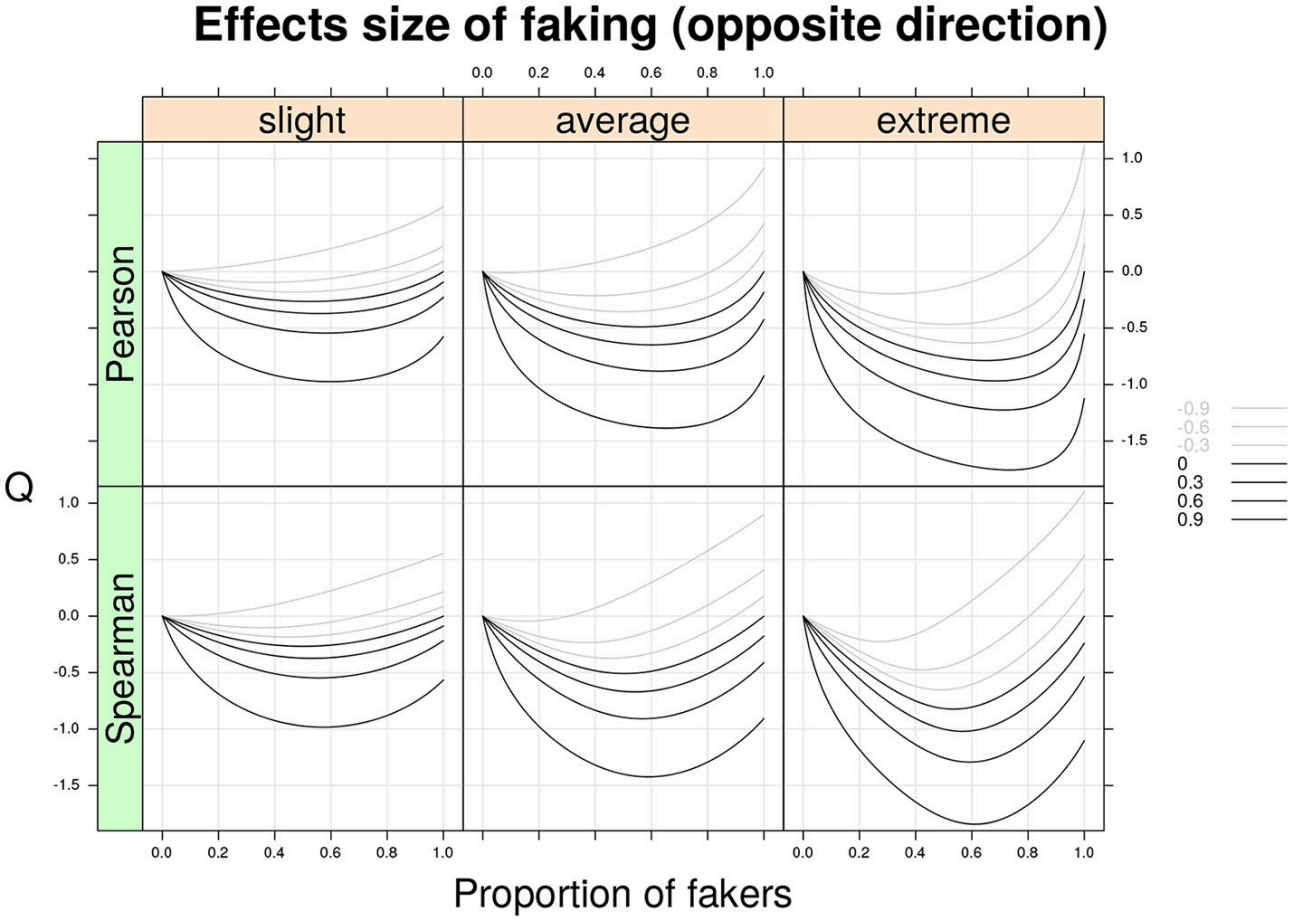
Cohen's *Q* statistic as a function of proportion of fakers in the population, type of faking model (slight, average, extreme), and original true correlation. The population analysis regards the two correlation indices (Pearson and Spearman) under the same model of faking but with opposite direction of faking for two discrete variables with common finite support (*v* = 5) and symmetric marginal distributions with mass probabilities 0.06, 0.25, 0.38, 0.25, and 0.06, respectively. Note the average model (*γ* = 3, *δ* = 3) represents a compromise between the slight model and the extreme model.

Figure 4 shows the *Q* statistic (computed separately for the two correlation indices) as a function of the same factors described earlier. However, this time the two variables *X* and *Y* are subjected to opposite directions of faking (e.g., *X* is corrupted by a faking good process, whereas *Y* is perturbed by a faking bad process, or vice versa). By a quick inspection of Figure 4, it is clear that the patterns of this second graphical representation mirror those shown in Figure 3. In general, for opposite models of faking with original non-negative correlations, the Cohen's *Q* statistic takes on negative values. For example, for an original positive Spearman correlation ρ_*d*_ = 0.6 and an average faking model with 20% of fakers, we observe a faking bias of *Q* = −0.6.

**Figure 5:**
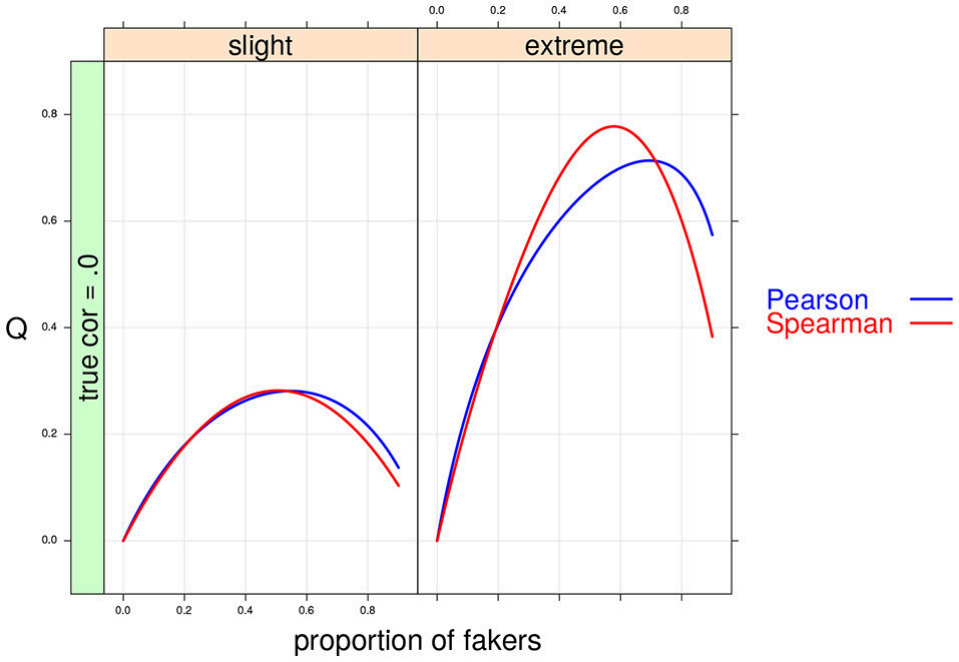
Cohen's *Q* statistic as a function of proportion of fakers and type of faking models. In this example, ρ_*d*_ = 0, *v* = 5 and same direction of faking are represented.

**Figure 6:**
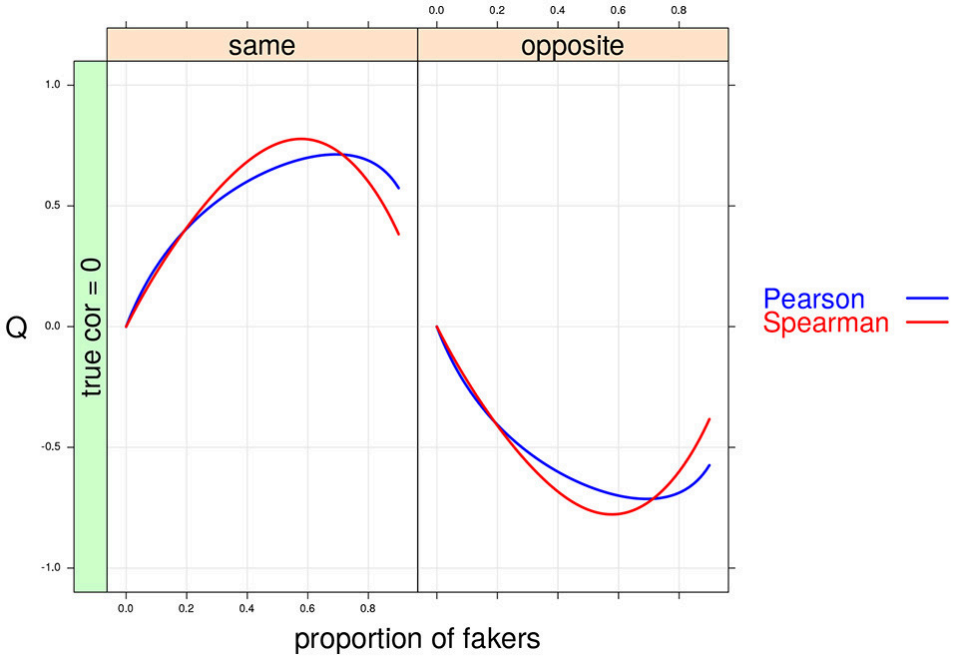
Cohen's *Q* statistic as a function of proportion of fakers and direction of faking in the two variables. In this example, ρ_*d*_ = 0, *v* = 5 and an extreme model of faking are presented.

**Figure 7:**
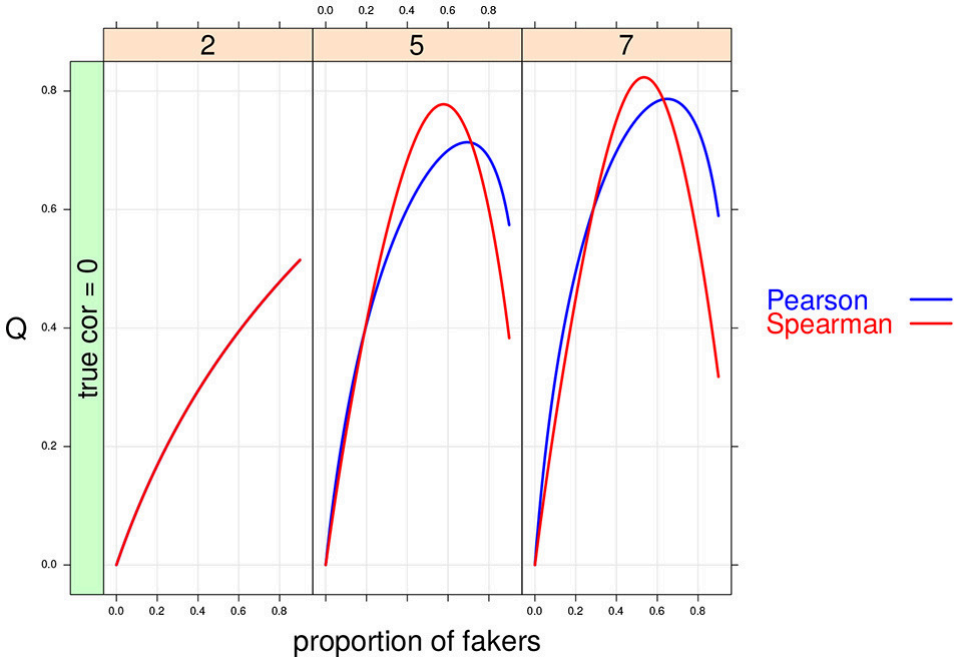
Cohen's *Q* statistic as a function of proportion of fakers and size of the support for the discrete variables (at three levels: 2, 5, 7). In this example, ρ_*d*_ = 0, and an extreme faking model with same direction are presented. Note that in the first panel (*v* = 2) the two correlation indices are completely superimposed.

**Figure 8:**
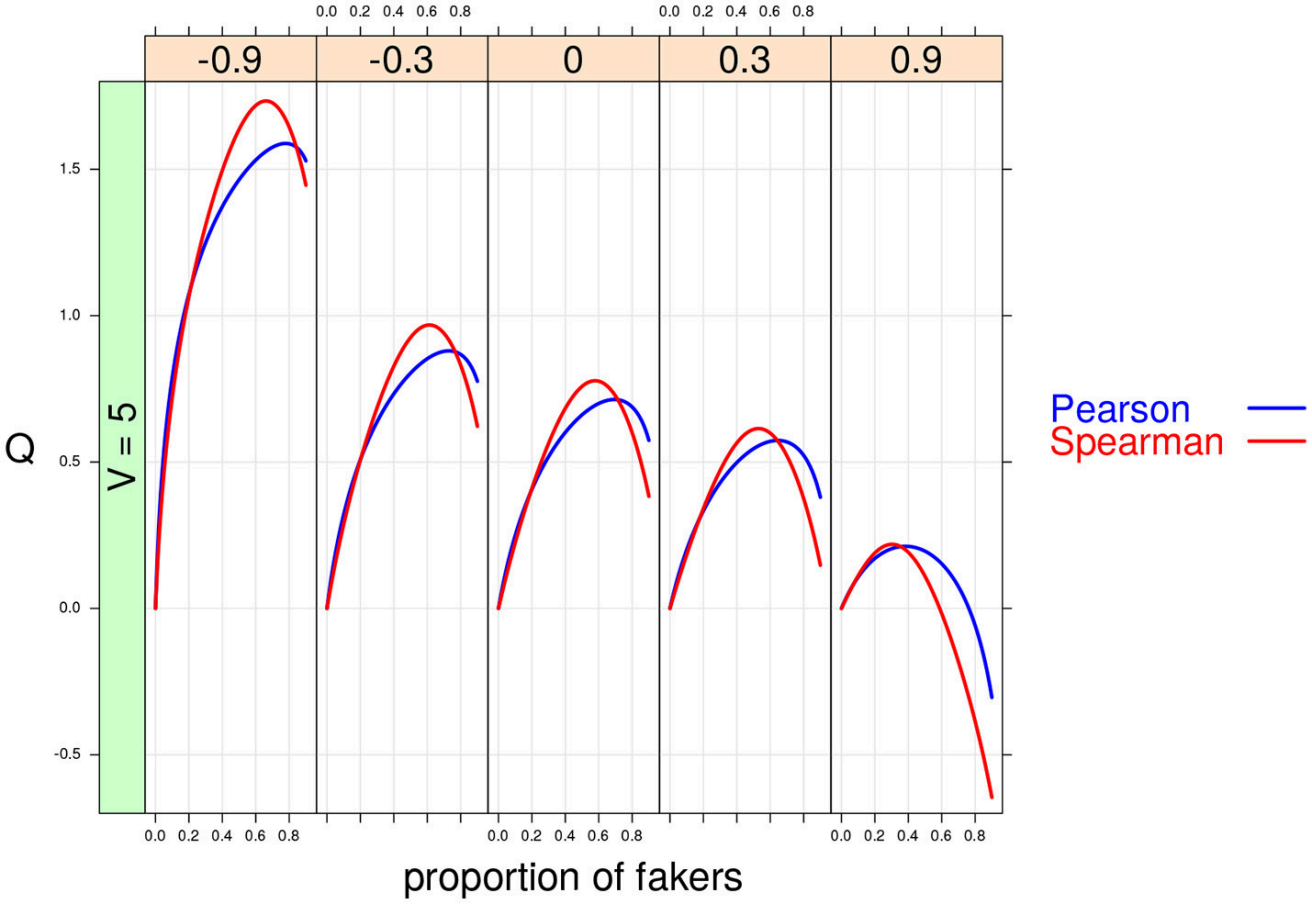
Cohen's *Q* statistic as a function of proportion of fakers and original correlation (at five levels: –0.9, –0.3, 0, 0.3, 0.9). In this example, *v* = 5, and an extreme faking model with same direction are presented.

Finally, Figures 5–8 provide more detailed representations which zoom in specific cross-combinations of factors' levels to highlight some relevant differences between the two correlation indices.

In sum, the graphical analysis clearly shows how a faking response process can substantially modify the original population correlation values by increasing (resp. decreasing) the strength of association depending on the quantity and quality of the perturbation mechanism. This clearly shows how faking manipulations can definitively have an impact, in a predictable manner, on the final mixture correlation.

## 5. Simulation study

The population analysis represents an elegant and efficient way to study the impact of faking on bivariate associations of discrete variables with finite supports. However, one of the main limitations of this kind of analysis is that it does not account for sample size effects on the evaluation of the faking mechanism. To overcome this shortcoming, in the following section we will describe a simulation study which extends the main results of the population analysis by considering some additional factors like sample size and different number of response levels for the discrete variables involved in the correlation.

### 5.1. Simulation design and data condition

We used the sample version *q* of the effect size statistic *Q* as the main dependent variable of the simulation design:

(15) $$\begin{array}{r} q=\frac{1}{2}\log\frac{1+r_{m}}{1-r_{m}}-\frac{1}{2}\log\frac{1+r_{d}}{1-r_{d}} \end{array}$$

with *r*_*d*_ and *r*_*m*_ being the sample correlations computed according to formulas (1) and (3), respectively. Moreover, six factors were systematically varied in a complete six-factor design:

- the sample size (*N*), at four levels: 20, 50, 100, 1,000;
- the proportion of fakers in the perturbed sample (*A*), at ten levels: 0, 0.10, …, 0.90;
- the type of faking model (*M*), at two levels: *slight* faking and *extreme* faking;
- the direction of faking (*DIR*), at two levels: *same* and *opposite*. A same-type perturbation is obtained whenever the two variables in the fake pattern are subjected to the same faking good manipulations. An opposite-type perturbation requires that in the fake pattern the first variable is subjected to a faking good manipulation, whereas the second variable is perturbed with a faking bad manipulation;
- the number of response options in the discrete variables (*V*), at three levels: 2, 5 and 7 response options;
- the true population correlation *R*_*d*_, at seven levels: −.9, −.6, −.3, 0, .3, .6, .9.

Let *n*, *α*, *m, dir, v*, and ρ_*d*_ be distinct levels of the factors *N, A, M, DIR, V* and *R*_*d*_, respectively. The following procedural steps were repeated 2,000 times for each of the 3, 360 = 4 × 10 × 2 × 2 × 3 × 7 combinations of factor levels (*n*, *α*, *m, dir, v*, ρ_*d*_) of the simulation design:

1. Generate a bivariate raw-data set **D** with size *n* according to the population correlation (ρ_*d*_). The data generation is performed using a standard MC procedure based on multivariate normal data (Kaiser and Dickman, 1962). More specifically, each row of **D** is sampled from the bivariate normal distribution *ϕ*(0, Σ_*d*_) with Σ_*d*_ being the correlation matrix with off-diagonal elements set to ρ_*d*_.
2. Discretize **D** on the basis of *v*−1 different thresholds by using the well-known method described by Jöreskog and Sörbom (1996) ^2^.
3. For each observation *s* = 1, …, *n*, sample a dichotomous (0/1) value *a* on the basis of proportion of fakers *α*. If *a* = 1, then replace the *s*−*th* row of **D** with the new row (replaced row) obtained using the model of faking *m* with direction *d*; otherwise keep the original *s*−*th* row of **D**. This procedure results in a mixture matrix **X**.
4. Compute and save the sample *q* effect size for the two correlation indices (Pearson correlation and Spearman correlation).

The whole procedure generated a total of 6, 720, 000 = 2, 000 × 4 × 10 × 2 × 2 × 3 × 7 matrices (**D**, **S**) as well as an equivalent number of pairs of correlation coefficient estimates for each of the two types of matrices^3^.

### 5.2. Results

**Figure 9:**
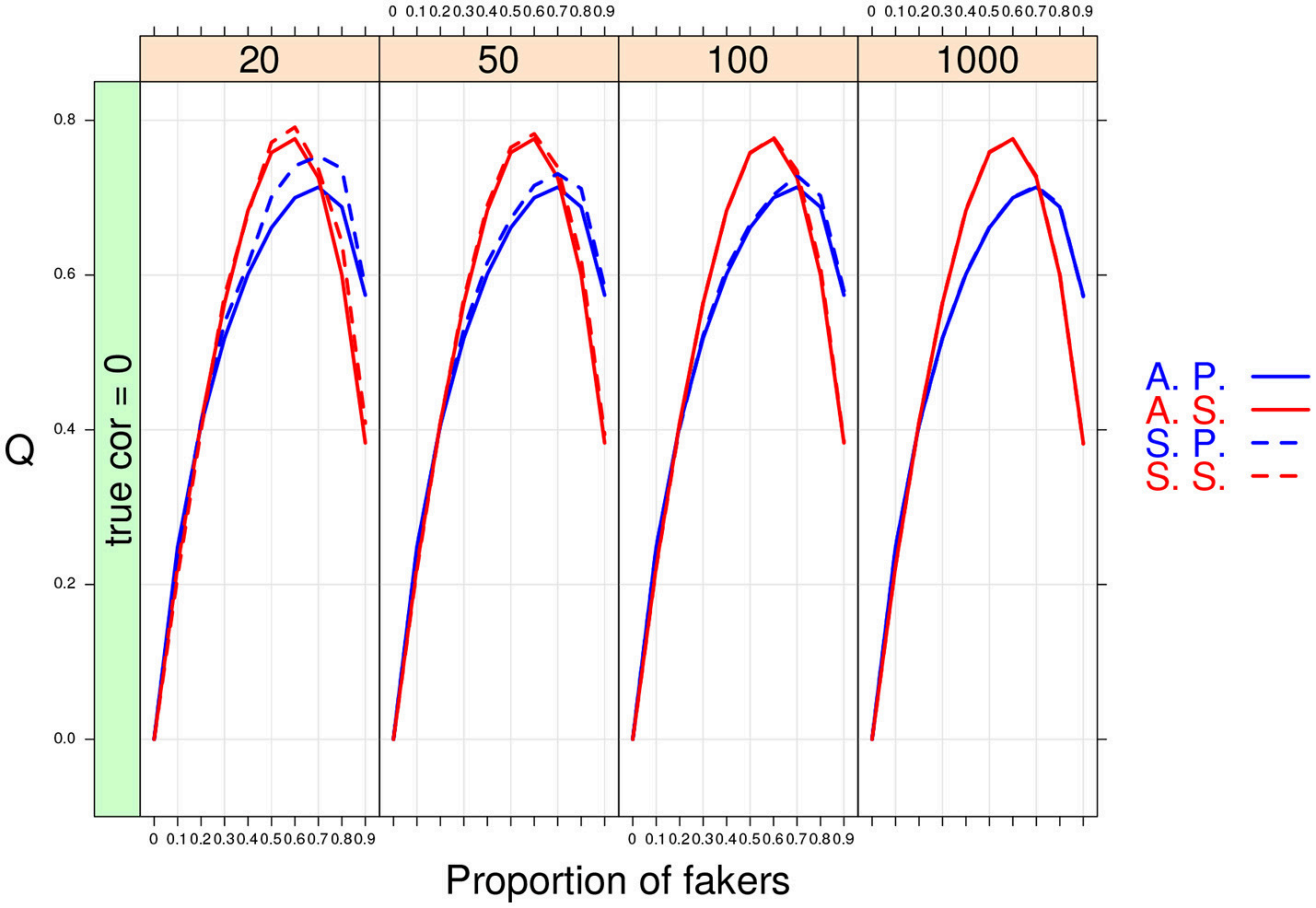
Cohen's *Q* statistic as a function of proportion of fakers and sample size (at four levels: 20, 50, 100, 1,000). In this example, ρ_*d*_ = 0, *v* = 5, and an extreme faking model with same direction are presented. A.P. and A.S. stand for Asymptotical Pearson's correlation and Asymptotical Spearman's correlation, respectively. S.P. and S.S. stand for M.C. Pearson's correlation and M.C. Spearman's correlation, respectively.

As expected the MC results converged to the population ones when large sample sizes were considered in the simulation design. However, the overall performance of the two association indices was different under small sample size conditions. In particular, the non-parametric correlation outperformed the product-moment correlation (see Figure 9) as its sample estimates were closer to the population values even with very small sample sizes (e.g., *n* = 20).

Tables 1–3 report the median values of the sample *q* statistic for the faking slight model and the faking extreme model with same direction, respectively. For the sake of space the three tables only represent the median values of the *q* statistic averaged across the four distinct sample size levels (20, 50, 100, 1,000) using the Spearman correlation only (which was the most sensitive correlational measure to faking perturbation). Just like for the population analysis, the empirical effect size statistic is also more affected by the presence of extreme faking manipulations in the sample, and the magnitude of this effect clearly depends on the original value of the correlation. Also the factor *DIR* modulates the observed pattern of the *q* statistic. In particular, the *same* direction of faking for both variables increases (on average) the final correlation, whereas the *opposite* direction of faking decreases (on average) the final perturbed correlation. This effect is consistent across all the combinations of levels of factors involved in the simulation design, unless we consider polytomous variables and very high positive original correlations (e.g., 0.9) for which a negative effect of *q* can be observed (Tables 2, 3, last row).

**Table 1:** Effect size *q* as a function of percentage of fakers, original correlation, and the type of faking model.

***R*_*d*_**	**Slight**						**Extreme**					

	**10%**	**20%**	**40%**	**60%**	**80%**	**90%**	**10%**	**20%**	**40%**	**60%**	**80%**	**90%**
−0.9	0.269	0.440	0.672	0.831	0.955	0.986	0.263	0.436	0.676	0.832	0.946	0.992
−0.6	0.146	0.260	0.430	0.558	0.659	0.709	0.145	0.255	0.430	0.556	0.665	0.700
−0.3	0.110	0.201	0.347	0.457	0.557	0.594	0.110	0.203	0.349	0.460	0.555	0.594
0	0.089	0.167	0.298	0.400	0.483	0.523	0.090	0.167	0.298	0.396	0.474	0.521
0.3	0.075	0.141	0.255	0.359	0.432	0.491	0.075	0.145	0.256	0.352	0.428	0.481
0.6	0.064	0.121	0.233	0.316	0.398	0.451	0.062	0.123	0.224	0.317	0.394	0.432
0.9	0.047	0.098	0.192	0.278	0.343	0.372	0.049	0.102	0.189	0.263	0.363	0.350

*Binary variable case: V = 2 with same direction of faking*.

**Table 2:** Effect size *q* as a function of percentage of fakers, original correlation, and the type of faking model.

***R*_*d*_**	**Slight**						**Extreme**					

	**10%**	**20%**	**40%**	**60%**	**80%**	**90%**	**10%**	**20%**	**40%**	**60%**	**80%**	**90%**
−0.9	0.433	0.654	0.873	0.931	0.839	0.712	0.753	1.078	1.436	1.630	1.627	1.497
−0.6	0.213	0.353	0.510	0.546	0.469	0.363	0.427	0.681	0.991	1.153	1.122	0.987
−0.3	0.138	0.241	0.359	0.382	0.307	0.218	0.306	0.507	0.780	0.911	0.852	0.699
0	0.101	0.178	0.268	0.277	0.201	0.118	0.235	0.404	0.641	0.738	0.658	0.480
0.3	0.078	0.137	0.198	0.189	0.110	0.029	0.190	0.340	0.534	0.584	0.456	0.261
0.6	0.057	0.097	0.132	0.101	0.001	−0.076	0.153	0.274	0.418	0.411	0.223	−0.008
0.9	0.023	0.026	−0.008	−0.096	−0.238	−0.324	0.108	0.179	0.209	0.083	−0.230	−0.499

*Ordinal variable case: V = 5 with same direction of faking*.

**Table 3:** Effect size *q* as a function of percentage of fakers, original correlation, and the type of faking model.

***R*_*d*_**	**Slight**						**Extreme**					
	**10%**	**20%**	**40%**	**60%**	**80%**	**90%**	**10%**	**20%**	**40%**	**60%**	**80%**	**90%**
−0.9	0.475	0.701	0.926	0.980	0.893	0.780	0.900	1.246	1.621	1.782	1.727	1.562
−0.6	0.214	0.358	0.507	0.544	0.468	0.373	0.499	0.777	1.105	1.249	1.159	0.989
−0.3	0.137	0.236	0.348	0.372	0.297	0.211	0.357	0.590	0.882	0.991	0.873	0.691
0	0.097	0.172	0.255	0.260	0.181	0.107	0.278	0.470	0.724	0.800	0.652	0.449
0.3	0.071	0.128	0.177	0.165	0.083	0.010	0.221	0.395	0.595	0.622	0.432	0.210
0.6	0.049	0.078	0.103	0.058	−0.043	−0.120	0.180	0.317	0.462	0.411	0.164	−0.079
0.9	0.002	−0.021	−0.094	−0.213	−0.356	−0.454	0.123	0.197	0.183	−0.005	−0.358	−0.633

*Ordinal variable case: V = 7 with same direction of faking*.

For the factor *V*, we observe an interaction effect with the proportion of fakers *A*. In particular, for dichotomous variables we observe a positive monotonic relation between the amount of fakers in the sample and the effect size *q*. By contrast, a concave relationship results when polytomous variables (*V* = 5 or *V* = 7) are considered (see also Figure 9). Finally, for polytomous variables with negative original correlations, the biggest effect of faking is observed when there are 60% of fakers in the sample (*q*>0.4).

Another interesting effect can be observed for the factor *R*_*d*_. Under faking manipulations, low original correlations (e.g., |ρ_*d*_| ≤ 0.3) may be biased both in terms of observed magnitude as well as correlation sign. For example, an original correlation ρ_*d*_ = −0.3 can be transformed into a final positive correlation when a medium to large effect size *q* is present (e.g., Table 2, cell corresponding to extreme model with 20% of fakers).

**Figure 10:**
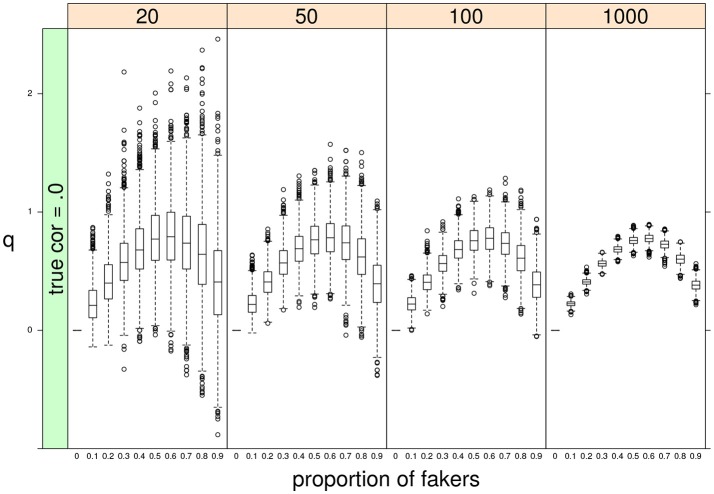
Boxplots of sample *q* statistic as a function of proportion of fakers and sample size (at four levels: 20, 50, 100, 1,000).In this example, *q* was computed for the Spearman correlation only. ρ_*d*_ = 0, *v* = 5, and an extreme faking model with same direction are presented.

Finally, some important results pertain the effect of sample size *N* or the proportion of fakers *A* on the variability of *q*. As expected, a negative relationship occurs between *N* and the variance of *q*: the lower the sample size, the bigger the variability of the effect size *q* (see Figure 10). By contrast, we observe a positive association between *A* and *q*: the larger is the proportion of fakers in the sample, the wider is the variance of *q* (see Figure 10). In general, we may notice that increasing sample size does not substantially modify the trend of the sample *q* statistic which keeps the same pattern across the four distinct levels of the sample size factor. Another interesting effect is the concave relationship with polytomous items that is also stable and consistent across all the four sample sizes. This effect is particularly evident if we consider original true null correlations (Figure 10).

In what follows, we provide a possible answer to explain why an extreme faking model disposes the computed correlations to be affected more by larger levels of faking perturbations in the simulated data. A very clear and simple result was that, on the one side, the correlation values decreased by increasing levels of fake observations in the data, as this usually tends to weaken the original true relationship between the two variables. On the other side, the observed correlations followed a hump shaped curve as a function of increasing amount of faking perturbation. Noticeably, the largest variances were observed for proportion of fakers close to 50–60%, whereas the lowest variances occurred at the lowest levels of fake observations. However, when the proportion of fakers (in the extreme faking model) is high, the majority of replaced values will be closer to the upper bound *V* = 7 and the corresponding variances will tend toward a minimal value. This effect is particularly evident in discrete variables with a limited support.

Very similar results were observed for more complex multivariate data analysis models (e.g., Lombardi and Pastore, 2012; Pastore and Lombardi, 2014).

## 6. Brief discussion and conclusions

The main motivation of this article was to point out the impact of fake data threats when analyzing self-report measures. We limited our study to the evaluation of fake data on two simple bivariate measures of association: (a) the product-moment correlation (b) the Spearman's rank based correlation.

The results of our population-level analysis and MC simulation highlighted the way the impact of faking (on the association between two variables) is affected mostly by the specific model of faking adopted to manipulate the original data. In other words, it depends mainly on how much the faking process changes the marginal means of the two variables. As expected, the extreme faking model has a greater impact on the location parameter as compared to the slight faking model and this also entails a bigger effect on the variances of the two variables and consequently on the final observed correlation.

The main result of our SGR analysis is that it is possible, under certain faking conditions, to either increase or decrease an original correlation in a predictable and systematic way. From an applied perspective, this general result may have many relevant implications. For example, in some sensitive contexts statistically significant correlations could easily be due to false positive associations (spurious correlations) resulting from structured faking manipulations. On the contrary, nonsignificant correlations may, instead, reflect true associations which have been corrupted by some kind of destructuring faking process due protection mechanisms adopted by the respondents.

The most dangerous effects of faking manipulation are observed for small or medium correlations as even small effects of faking could potentially change the observed *p*-values of a correlation analysis. Furthermore, like true extreme correlations are sensitive to fake data as they are transformed in milder correlations, true small correlations are also affected by faking manipulations in both directions (reflecting either positive or negative associations) in the final correlation. The latter implies that faking manipulations could easily reverse the sign of an original correlation (*sign effect* of faking).

In our study, we did not perform a direct evaluation of the significance level of the corrupted correlation result, instead we limited the analysis to the impact of faking on the size of the correlational values only. However, a systematic analysis of the modulation of the significance level on the correlational analysis could be easily obtained by slightly changing the structure of the simulation design. For example, for each cell and each repetition in the simulation design we could compute the corresponding *p*-values for the honest correlation and fake correlation and construct a two by two frequency table reporting (a) the number of jointly significant results for the two conditions (b) the number of jointly nonsignificant results for the two conditions (c) the number of diverging results (significant for the honest condition and non significant for the faking condition) (d) the number of diverging results (nonsignificant for the honest condition and significant for the faking condition). The relative proportions of this classification table would inform us about the effect of faking on the significance results.

In this contribution, we also showed how the SGR paradigm can be used to predict the behavior of the correlation indices under different faking perturbations. In particular, we presented an extension of the SGR representation which is based on a mixture model which is more consistent with the way individuals may actually fake their responses to self report measures. We also provided a population representation of the faking problem by directly modeling the population probability distributions which define the tokens of the SGR paradigm.

As with other Monte Carlo studies, our investigation involves simplifying assumptions that may result in lower external validity such as, for example, the assumption that the threshold values in the honest generative model are considered invariant across the variables and symmetric around the mean of the bivariate standardized distribution. Unfortunately, this restriction clearly limits the range of empirical faking processes that can be mimicked by using our approach. However, it is not difficult to modify the sampling process to guarantee that asymmetric distributions apply for the data generation process according to the true original model. In this regard, we make available to the readers the full R code to run the population analyses that take into account diverse distributional properties for the joint distribution **D** (e.g., for skewed distributions, TO ADD URL).

Moreover, a straightforward extension of the SGR modeling would pertain individual differences in the faking response profile which goes beyond the simple binary categorical representation (honest individuals vs fakers) provided here. More specifically, we could mimic unequal values for the faking parameters as a function of some respondent's characteristics or features in the mixture model. In this generalization we could use *c* different categories of faking response patterns as follows:

(16) $$\begin{array}{rcl} s_{xy} & = & \left(1-\alpha^{*}\right)p_{xy}+\overset{c}{\underset{l=1}{\sum }}\alpha_{l}z_{xy}^{\left(l\right)},\;\left(x,y\right)\in {\left\{1,2,\ldots ,v\right\}}^{2} \end{array}$$

where *s*_*xy*_ is the mixture probability of the observed variables *X* = *x* and *Y* = *y*. In Equation (16), parameter *α*_*l*_ ∈ [0, 1] denotes the proportion of type-*l* fakers in the population such that

(17) $$\begin{array}{r} 0\leq \left(\alpha^{*}=\overset{c}{\underset{l=1}{\sum }}\alpha_{l}\right)\leq 1. \end{array}$$

For example, we could model three different types (*c* = 3) of faking behavior (e.g., slight, average, and extreme) in the population with the corresponding proportions. This would allow us to make use of additional information related to the individual's characteristics (e.g., desirability measures) in order to derive the faking parameters of the replacement model as a direct function of these observed characteristics.

Finally, for empirical applications specific hypotheses about the data modeling (both true model and replacement distribution) could be derived from previous studies, by adopting a Bayesian-type perspective about, for example, the setting of prior distributions on the basis of former empirical observations. Similarly, specific assumptions about the nature of the association could also be extracted from published norms of specific psychometric measurements (e.g., when psychological batteries are administered), or using explicit knowledge in line with personality theories or motivation theories. Overall, our main practical “guideline” would be that of encouraging data analysts and researchers to routinely include a plot showing the potential impact of the percentage of fakers on the correlation coefficients. This would be important for all those situations were an investigator is collecting data about sensitive topics where the specific amount of fakers cannot be known in advance. In this respect it is important to understand that the SGR methodology is fundamentally characterized by an interpretation-type perspective which tries to incorporate in a single global model representation both empirical and hypothetical information regarding the faking mechanism. In particular, we further stress that our proposal is not a way to detect faking at the individual or group level but, instead, a rational procedure which allows to critically evaluate the observed statistical results as if they were potentially corrupted or manipulated by faking mechanisms. In sum, we understand our proposal to be very similar to a sensitivity (or uncertainty) type analysis: if a researcher is interested in a correlation, then he/she should be “aware” that the observed correlation may be biased due to the presence of fakers and the faking mechanism.

However, it is important to highlight that faking is only one of the many examples of data manipulations that can affect the statistical results in self-report measures (e.g., cheating or guessing, random, extreme or reverse answering). Therefore future studies should also consider the interaction between these different sources of data manipulations on the final correlation results.

## Author contributions

All authors listed have made a substantial, direct and intellectual contribution to the work, and approved it for publication.

### Conflict of interest statement

The authors declare that the research was conducted in the absence of any commercial or financial relationships that could be construed as a potential conflict of interest.

## Footnotes

^1^For the sake of simplicity in our representation we assume that both variables share the same support. However, a natural extension to the case with unequal supports is straightforward and will not be discussed here.

^2^In the simulation study all the continuous data set **D** were discretized into symmetrically distributed variables with the following point mass values: 0.5, 0.5 for *v* = 2; 0.06, 0.25, 0.38, 0.25, 0.06, for *v* = 5; 0.02, 0.09, 0.23, 0.31, 0.23, 0.09, 0.02, for *v* = 7.

^3^In step 3 (resp. step 5), the sample **D** (resp. **X**) was re-sampled whenever the computation of one of the two association indices yielded unfeasible values (e.g., because one column of the data matrix had zero variance). However, the percentage of unfeasible solutions was very low, about 0.035% of the total 6, 720, 000 samples.
